# Class and isotype of VlsE-specific antibody differentiates Lyme disease stage

**DOI:** 10.1128/jcm.00347-25

**Published:** 2025-07-15

**Authors:** Nisha Nair, Adriana Marques, Elizabeth J. Horn, Grant Brown, Maria Gomes-Solecki

**Affiliations:** 1 Department of Microbiology, Immunology and Biochemistry, University of Tennessee Health Science Center 274062, Memphis, Tennessee, USA; 2 Lyme Disease Studies Unit, Laboratory of Clinical Immunology and Microbiology, National Institute of Allergy and Infectious Diseases, National Institute of Health 35037, Bethesda, Maryland, USA; 3 Lyme Disease Biobank, Portland, Oregon, USA; 4 Department of Biostatistics, University of Iowa 366203, Iowa City, Iowa, USA; 5 Immuno Technologies Inc, Memphis, Tennessee, USA; Mayo Clinic Minnesota, Rochester, Minnesota, USA

**Keywords:** lyme disease, erythema chronicum migrans, PTLDS, time, disease progression, early diagnosis, antibody, immunoglobulin isotypes, immunoglobulin class switching, VlsE, *Borrelia burgdorferi*

## Abstract

Establishment of immunoglobulin diversity is contingent on recombination that occurs both at the Fab and at the Fc regions of the immunoglobulin, and this process is time dependent. Based on this principle, we questioned whether Lyme disease stage can be distinguished by quantification of immunoglobulin class and IgG isotype specific to VlsE in serum from clinically characterized patients. We used an enzyme immunoassay to categorize serologic antibodies to VlsE antigen as well as machine-learning techniques to train and integrate multiple predictors to identify likely disease stage. We found that IgM/IgG3/IgG1/IgA1 was enriched in serum obtained in the earliest stages, whereas IgG3/IgG1/IgG4 was enriched in Lyme arthritis. IgG2 detection was unremarkable across all disease stages. Post-Treatment Lyme Disease Syndrome (PTLDS) serum was enriched in IgG3/IgG1/IgA1 but lacked IgM. The multivariable models showed better predictive accuracy than any single immunoglobulin model, with more than half of panels perfectly identified by random forest under cross validation (56%) vs a maximum of 38% for a model using IgG1 alone. The findings suggest a characteristic succession of VlsE-specific antibody switching between immunoglobulin class and IgG isotype as Lyme disease progresses from early to late stages. The data also suggest that immunoglobulin class and IgG isotyping are likely more helpful to distinguish early Lyme disease cases. Comprehensive evaluation of immunoglobulin class (M, G, A) and IgG isotypes (1/2/3/4) provides time-dependent pathogen-induced host response information to current Lyme disease antibody detection and may be useful for differentiation of disease stage.

**IMPORTANCE:**

The order of switching between the immunoglobulin heavy chain (Fc) is time dependent, progressing from IgM/D to IgG3/IgG1/IgA1/IgG2/IgG4 and later to IgE/IgA2. In this study, we show that *B. burgdorferi*-VlsE-specific antibody switching proceeds in a predictable sequence between class (Ig M/G/A) and IgG isotype (IgG 1/2/3/4) as Lyme disease progresses from early to late stage and that antibody class and isotype may be more helpful to distinguish the early stages of Lyme disease. This study advances our understanding of the tempo and structure of the humoral immune response to *B. burgdorferi* and is applicable to the development of new diagnostic assays for Lyme disease.

## INTRODUCTION

Lyme disease is the most common vector-borne disease in the USA, with approximately 476,000 people diagnosed and treated annually (1, 2). The infection starts after *Borrelia burgdorferi* is injected into the skin by the bite of an infected ixodid tick. The characteristic erythema migrans (EM) rash is the classic clinical sign of early localized infection and is the most common manifestation of Lyme disease. If untreated, spirochetes disseminate to distant sites causing multiple EM lesions, early Lyme neuroborreliosis, and carditis. Arthritis is the most common late manifestation of Lyme disease in the USA (3, 4). While most patients with Lyme disease will recover after treatment, a subset continues to have non-specific symptoms and is classified as post-treatment Lyme disease symptoms or syndrome (PTLDS). The mechanisms that underlie PTLDS are not known (5).

While EM is a clinical diagnosis, laboratory testing is recommended to support the diagnosis of other manifestations of Lyme disease. The US Center for Disease Control and Prevention (CDC) recommends a two-tiered approach using serologic testing (6–8). While these two-tier testing algorithms are specific and sensitive for later manifestations of Lyme disease (9, 10), they are relatively insensitive for the detection of early infection. Finding biomarkers associated with Lyme disease stage could help guide the development of new diagnostic assays especially for early disease when accurate diagnosis impacts successful treatment and prognosis. While current serodiagnostic algorithms allow for rough classification of disease stage based on IgM/IgG, with IgM representing acute cases up to 1 month (7), and all others classified under IgG, machine-learning models allow for finer granularity of predictable disease stage determinations.

**Fig 1:**
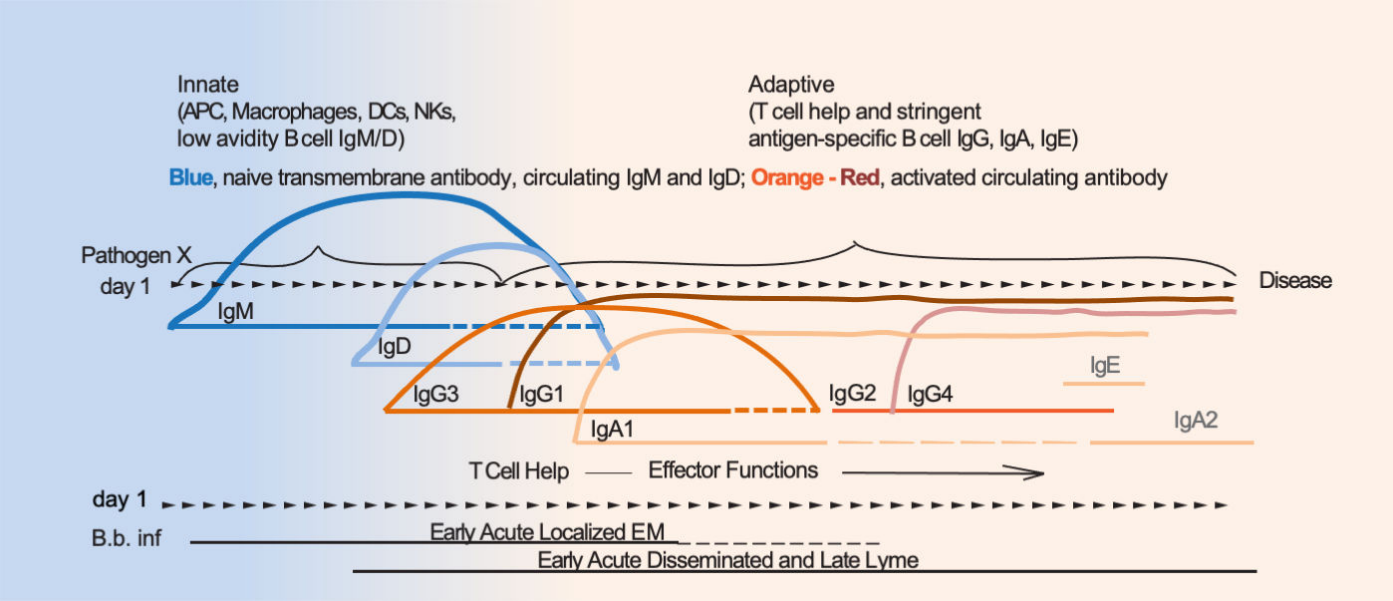
Hypothetical development of anti-*B*. *burgdorferi* antibody as Lyme disease progresses from early to late stage. Legend: APC, antigen presenting cell; DC, dendritic cell; NK, natural killer cell; Ig, immunoglobulin; EM, erythema migrans; B. b. inf, *Borrelia burgdorferi* infection.

Although human immunoglobulin G (IgG) subclasses were numbered according to its abundance in serum, the establishment of immunoglobulin diversity is hierarchical. Early in B cell development, immature B cells that have successfully produced an IgM B cell receptor extend transcription of the heavy chain locus to include the Cδ exons downstream of Cµ. Alternative splicing permits co-production of IgM and IgD. These now newly mature IgM + IgD + B cells share the same variable domains, leave the bone marrow, and enter the blood stream to migrate to other lymphoid tissues. Later during B cell development, and in response to antigenic stimulation and cytokine regulation, the same variable domains may associate with the other three antibody isotypes (IgG, IgA, and IgE) in a controlled process that reflects an adaptive immune response (11, 12). Thus, the presence of a particular antibody isotype in serum at a given point in time post infection reveals the type of immune response (innate, less specific opsonizing-IgM/IgD or adaptive, antigen-specific IgG, IgA, IgE) ongoing in the host against the challenging pathogen. Based on these fundamental principles, we questioned whether it is possible to improve current Lyme disease testing to distinguish disease stage by targeting the four immunoglobulin classes (IgM/D/G/A) and all four IgG isotypes (IgG1/2/3/4) (Fig. 1). To evaluate this hypothesis, we targeted VlsE, a protein expressed by *B. burgdorferi* at all stages of infection (13–15) and used extensively for the development of diagnostic assays for Lyme disease (16–20), to leverage the acquisition of antibody profiles to this antigen through disease progression. We performed statistical analyses of the data by examining the ability of multivariable models to predict disease stages based on the understanding that signatures may be the strongest when combining information across multiple immunoglobulin classes and isotypes.

## MATERIALS AND METHODS

### Human serum panels

A summary of the sera panels is provided in Table 1.

**TABLE 1:** Sera panels used in this study

Sera panel (*n*)	Source	Details
Early + Late Lyme (*n* = 40)	Center for Disease Control and Prevention	Included serum from clinically characterized early Lyme (*n* = 25) and late Lyme (*n* = 10) patients and healthy (*n* = 5)
Blinded (*n* = 47)	Lyme Disease Biobank	Blinded panel with samples from patients suspected of Lyme disease + healthy controls (~2016): some had erythema migrans, some had positive serology, two had microbiologic evidence of infection, and the healthy individuals were from the same endemic areas
Early Acute <1 mo (*n* = 32)	NY State Department of Health (NYSDOH32)	Patients suspected of having very early Lyme disease, C6 EIA positive plus IgM immunoblotting positive (Standard Two-Tier positive, C6+, IgM+)
Early Acute >1 mo (*n* = 26)	NY State Department of Health (NYSDOH26)	Patients suspected of having early Lyme disease, C6 EIA positive plus IgM/IgG immunoblotting positive (Standard Two-Tier positive C6+/IgM+ IgG+ )
Early Convalescent (*n* = 39)	NY State Department of Health (NYSDOH39)	Patients suspected of being in Convalescent phase (*n* = 39), C6 EIA positive plus IgG immunoblotting positive (Standard Two-Tier positive, C6+/IgG+)
Late Lyme (*n* = 18)	National Institutes of Health	Patients with Lyme arthritis
PTLDS (*n* = 25)	National Institutes of Health	Post treatment Lyme disease syndrome
Control (*n* = 40)	BioIVT	Samples from non-endemic region, no known Lyme disease

#### Early and late Lyme, Centers for Disease Control and Prevention (CDC), *n* = 40 samples

This panel contained 35 samples collected from patients diagnosed with Lyme disease by experienced physicians in endemic areas (Northeast and upper-Midwest). Five of the samples included in the panel were obtained from healthy individuals from the same areas. *B. burgdorferi* was cultured from 88% of the early Lyme cases, and the remaining patients met a rigorous case definition for early disseminated or late Lyme disease and had a seropositive ELISA result. This panel was provided by Dr. Martin Schriefer from the NCID/CDC in the late nineties.

#### Blinded, Lyme Disease Biobank (LDB), *n* = 47 samples

This panel contained serum samples provided by the Lyme Disease Biobank in ~2016 from patients from Eastern Long Island, NY, and Martha’s Vineyard, MA. Some patients had physician assessment of an erythematous expanding rash, some had serologic evidence of infection, and two had microbiological confirmation of Lyme disease (21); this panel also included samples from healthy individuals from the same endemic areas.

#### Early Lyme disease, New York State Department of Health (NYDSOH), *n* = 97 samples

Three sera panels were obtained from Dr. Susan Wong at the NY State Department of Health in 2017. These panels contained serum from patients who presented with an erythematous skin lesion consistent with EM and history of recent tick bite, *or* summer flu-like illness to clinics in endemic New York State that subsequently tested positive by Standard Two Tier (STT) serology (ELISA followed by Western Blot) at New York State Department of Health. The panels are defined as follows: NYSDOH32 contained 32 human serum samples tested as C6+/IgM+ (classified as Early Acute <1 month); NYSDOH26 contained 26 human serum samples tested as C6+/IgM+ IgG+ (classified as Early Acute >1 month); NYSDOH39 contained 39 human serum samples tested as C6+/IgG+ (classified as Early Convalescent).

#### Late Lyme and PTLDS, National Institutes of Health (NIH), *n* = 43 samples

Patients acquired the infection in the USA and fulfilled the 2017 CDC case definition of confirmed or probable Lyme disease (22). These included samples from 18 patients with Lyme arthritis and 25 patients classified as Post-Treatment Lyme Disease Syndrome. Samples were collected after the start of antibiotic therapy. Patients with Lyme arthritis presented with intermittent or persistent attacks of joint swelling, primarily in one or few large joints, particularly involving the knee. All patients were seropositive for *B. burgdorferi* antibodies by the standard two-tier criteria (23). Patients with PTLDS had Lyme disease, received a minimum of one course of recommended therapy, and had persistent or relapsing nonspecific symptoms that began within 6 months of treatment, which were severe enough to cause a reduction in activities (3, 24, 25).

#### Controls, BioIVT, *n* = 40 samples

Serum was purchased from a commercial source (BioIVT, MD) from healthy individuals from a non-endemic area without known Lyme disease.

### VlsE1 ELISA

Enzyme-linked immunosorbent assay (ELISA) was used to detect antibodies to VlsE antigen in serum. Recombinant *Borrelia burgdorferi* VlsE1 was sourced from PROSPEC (Israel). The VlsE1-ELISA was optimized using known negative and positive samples to identify the best secondary antibody dilution per immunoglobulin class and IgG isotype. A 96 well microtiter plate (Nunc MaxiSorp flat-bottom 96 well plate, ThermoFisher Scientific, TN, USA) was coated with 0.1 µg of VlsE antigen in 100 µL of 1× ELISA coating buffer (VWR, PA, USA) and incubated overnight at 4°C. The microtiter plate was washed with 300 µL of 1× wash buffer (Fisher Scientific, NH, USA) for 4 wash cycles using an ELISA plate washer (Fisher Scientific accuWash, NH, USA). The plate was blocked using 250 µL of blocking buffer (1% BSA in wash buffer) by incubating in a 37°C incubator for 2 h. Meanwhile, sera samples were thawed and diluted to 1:100 using blocking buffer (samples were run in duplicates). The plate was washed twice with 300 µL wash buffer followed by incubation with 100 µL of diluted sera at 37°C for 1 h. The plate was washed 4 times with 300 µL of 1× wash buffer followed by incubation with secondary antibody conjugated with HRP for 30 min at 37°C. The secondary antibodies and dilutions used for ELISA are the following: Mouse anti-Human IgG1 Fc Secondary Antibody, HRP (ThermoFisher Scientific, TN, USA, dilution 1:2,000); Mouse anti-Human IgG2 Secondary Antibody, HRP (ThermoFisher Scientific, TN, USA, dilution 1:1,000); Mouse anti-Human IgG3 Secondary Antibody, HRP (ThermoFisher Scientific, TN, USA, dilution 1:1,000); Mouse anti-Human IgG4 Fc Secondary Antibody, HRP (ThermoFisher Scientific, TN, USA, dilution 1:1,000); Mouse anti-Human IgD Secondary Antibody, HRP (Fisher Scientific, NH, USA, dilution 1:600); Peroxidase AffiniPure Goat Anti-Human IgM, Fc5μ fragment specific (Jackson ImmnunoResearch, PA, USA, dilution 1:75,000); and Mouse anti-Human IgA1 Fc Secondary Antibody, HRP (ThermoFisher Scientific, TN, USA, dilution 1:1,000). After the incubation with secondary antibody, the plate was washed for four cycles followed by incubation with 100 µL TMB Microwell Peroxidase Substrate (Fisher Scientific, NH, USA) at 37°C for 15 min. The reaction was stopped by adding 100 µL of TMB Stop solution (VWR, PA, USA). The plate was read on a spectrophotometer (SpectraMax) at 450 nm. The cutoff value was determined for each panel to categorize the OD_450_ value of a serum obtained as positive or negative. For analysis of clinical cohorts, each sample was tested in duplicate. The same five internal healthy controls were included in each plate to determine the cut-off (five standard deviations from the mean). Positive controls were not included in each plate to reduce sources for cross-contamination.

#### Statistical analysis using multivariable machine learning models

To examine the degree to which the serum panel labels could be recovered from the VlsE profiles, we focused on the panels of Lyme disease sera and one healthy control, and we considered VlsE responses to IgM, IgG3, IgG1, IgA1, and IgG4. We used two general approaches to predictive modeling. First, we employed single decision tree models, fit using the rpart package for R version 4.2.2 (26). These models provide an interpretable relationship between the outcome (in this case, serum panel) and the predictors (antibody responses), allowing visualization and straightforward summary of the patterns learned, while automatically exploring interactions and nonlinearities. To characterize the predictive performance of the model, we employed Leave-One-Out-Cross-Validation (LOOCV), and reported classification performance via a full confusion matrix, as well as estimated sensitivities, specificities, and positive and negative predictive values for identifying the sera panels. Classification tree tuning parameters were left at software defaults. Next, we explored the degree to which single decision trees sacrifice predictive power for explainability, employing several multivariable models for the same categorical outcome. We employed random forests (27), multinomial regression (28), penalized multinomial regression with pairwise interactions (29, 30), gradient boosted trees (31), and support vector machines (32). Each of these supervised learning techniques was again evaluated under Leave-One-Out-Cross-Validation (LOOCV), with performance characterized similarly. As before, we generally relied on default software parameters rather than extensive tuning; additional implementation details include an inner cross-validation loop for selecting the penalty term in glmnet, and an inner cross-validation loop for selecting the number of rounds of the boosting model (using a fixed tree depth of 4). Predictive performance is assessed in terms of the sensitivity, specificity, positive predictive value (PPV), and negative predictive value (NPV) for identifying each panel. We also present a table focused on case/control status in the supplemental material and provide a full example confusion matrix comparing observed panel labels and those predicted under cross-validation (e.g., while holding out observations to prevent overfitting) for random forests specifically. To provide a baseline for these predictive results, we also include pairwise Wilcoxon Rank Sum tests for each immunoglobulin between each pair of panels, adjusting within each table for multiple comparisons using the Benjamini Hochberg FDR method (33). Crucially, the predictive results give a key insight into the degree to which the immunoglobulin values can be used to distinguish among the panels, while the hypothesis test approach quantifies which panels and show evidence of a difference, on average, with respect to individual immunoglobulins, regardless of whether those significant differences are reliably predictive.

## RESULTS

### Immunoglobulin classes (M, G, A) and all IgG isotypes were detected in Lyme disease serum panels except IgG2 that was unremarkable

**Fig 2:**
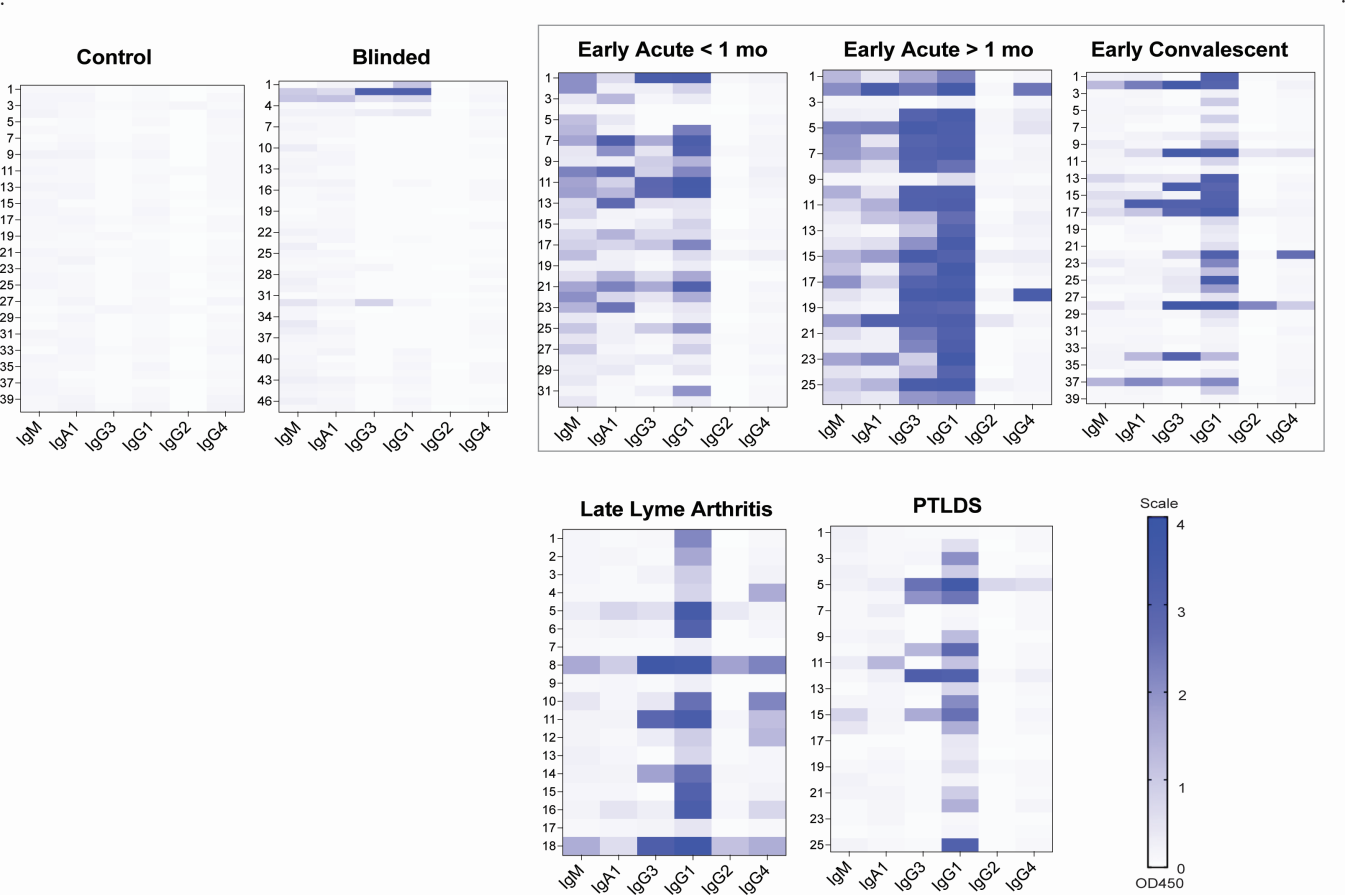
Heat map of VlsE-specific antibodies in Lyme disease patient sera classified by stage. The sera panels are defined as follows: Control, *n* = 40 samples from BioIVT; Blinded Lyme+Healthy, *n* = 47 samples from the Lyme Disease Biobank; Early Acute <1 month (mo), *n* = 32 samples from the New York State Department of Health; Early Acute >1 month (mo), *n* = 26 samples from the New York State Department of Health; Early Convalescent, *n* = 39 samples from the New York State Department of Health; Late Lyme arthritis, *n* = 18 samples from the National Institutes of Health; PTLDS, Post-Treatment Lyme Disease Symptoms, *n* = 25 samples from the National Institutes of Health. IgM, IgA1, and IgG isotypes (IgG1, IgG2, IgG3, IgG4) specific to *B. burgdorferi* VlsE was determined by ELISA. Scale 1–4, OD 450 nm.

**Fig 3:**
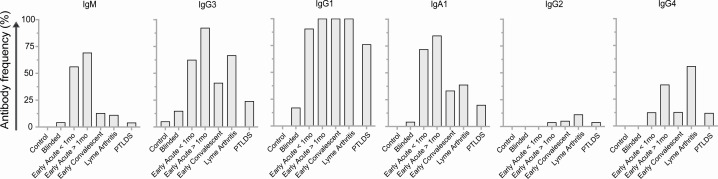
Proportion of antibody class and IgG isotype per Lyme disease stage. ELISA was used to determine OD450nm values for each serum sample against recombinant VlsE antigen.

Anti-VlsE IgM, IgG3, IgG1, IgA1, IgG2, IgG4 antibodies were tested in suspected and defined LD and in healthy sera panels using ELISA. The raw OD results are represented as heat maps in Fig. 2 for a bird’s-eye overview of the data, and the proportion of samples positive for VlsE1 is shown in Fig. 3. As expected for serologic analysis of antibody to *B. burgdorferi* antigen, the Early Acute >1 mo stage produced the strongest visual presence of 4 (IgM, IgG3, IgG1, IgA1) of the 6 immunoglobulins tested (Fig. 2). Analysis of antibody class and IgG isotype frequency per disease stage (Fig. 3) showed the expected pattern for IgM, a less specific immunoglobulin, which was enriched (56%–69%) in the serum panels classified as earliest disease (Early Acute). IgG3 to VlsE1 was mostly associated with Early Acute (63%–92%) and Lyme Arthritis (67%). IgG1 was the predominant antibody isotype in the IgG class across all stages of Lyme disease, being detected in 91% of Early Acute <1 month, 100% of cases > 1 month (Early Acute, Convalescent, Lyme Arthritis), and 76% of PTLDS. IgA1 was enriched in Early Acute (72%–85%), and IgG4 was enriched in Lyme Arthritis (56%). The detection of IgG2 to VlsE1 was negligible in most disease stages except in Lyme arthritis, which remained low at 11%.

### Presence of IgD class in Lyme disease serum

We evaluated IgD in a panel of sera containing early and late Lyme disease patient samples (CDC) and quantified the number of patients with more than 10 ng IgD per mL of serum (Fig. S1). We found that total IgD was present in early Lyme disease patients at a higher prevalence (55%) than late Lyme arthritis (30%). However, when we tested for the presence of IgD specific to VlsE in the Early Acute and Early Convalescent panels, the results were inconclusive.

### Determination of Lyme disease stage using a machine learning multivariate approach

**Fig 4:**
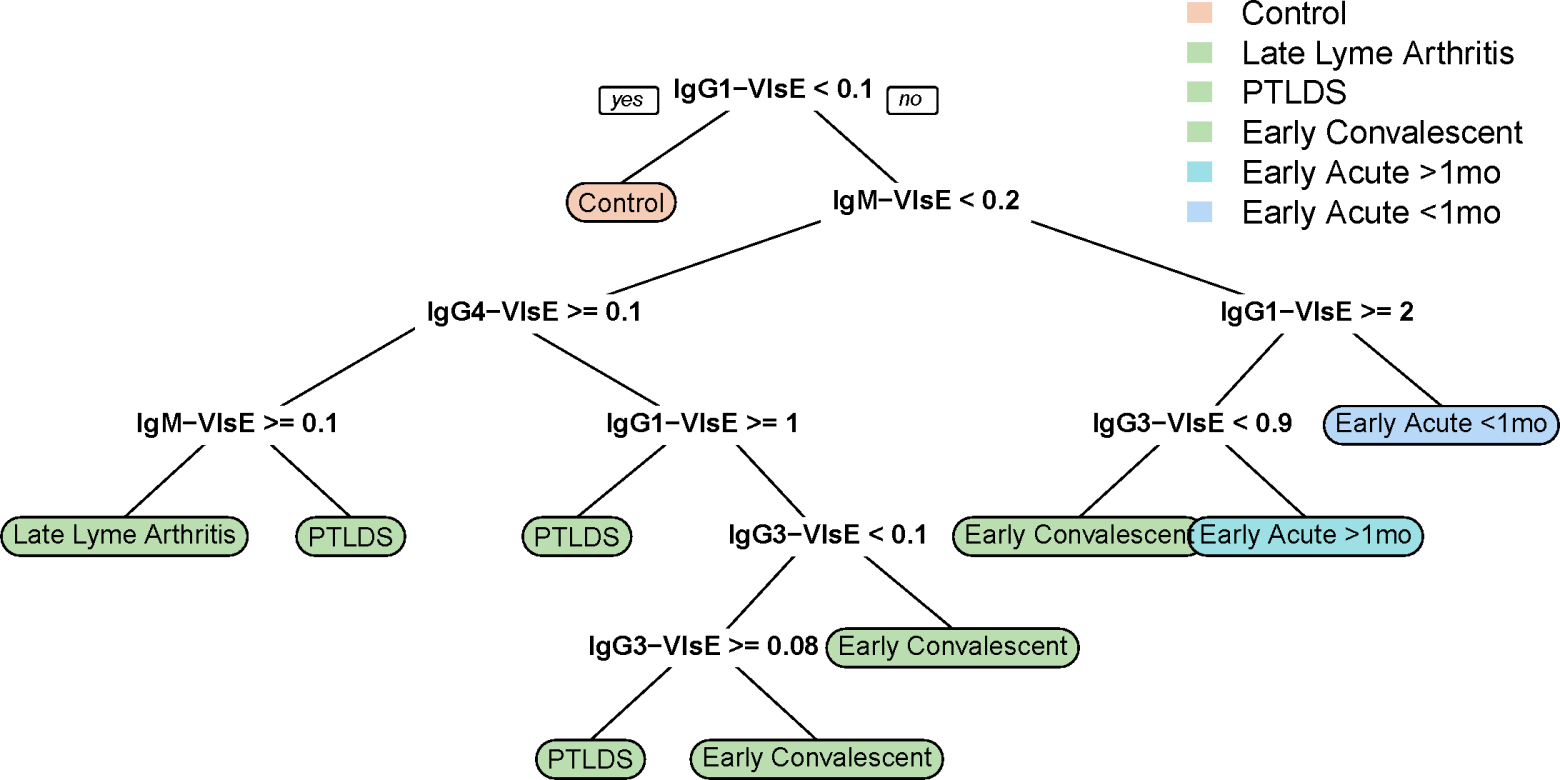
Decision tree model classifying disease stage from antibody class and isotype. Starting at the root, predictions are made by moving left or right down the tree into the terminal nodes, according to the comparisons outlined in each split.

Evaluating the patterns learned by the multivariable approaches, we see that the decision tree model tended to employ IgG1 first to sort out healthy controls and then to differentiate based on IgM levels to broadly group earlier and later panels (Fig. 4). The Blinded panel was excluded from the multivariate analysis because it contained samples from healthy individuals. The more nuanced splits employ a mix of all the antibodies included except IgA1 (which nevertheless is used somewhat in the ensemble models). Figure 4 highlights the pattern learned by the model, showing how a predicted serum panel is motivated from the antibody features. Decisions as to how to classify an observation as a particular serum panel are made by traversing the tree from top to bottom, checking the immunogloblulin cutoff noted and traversing left or right accordingly. The leaf nodes of the tree describe the final prediction. In Table 2, the predictive characteristics of the various models described are shown for the six sera panels. In general, as expected, the chosen immunoglobulins are particularly effective for classification of early Lyme disease, though in a larger experiment, these methods could be extended to examine antigens which inform groups like Lyme Arthritis and PTLDS. This distinction is also clear in Table 3, which shows the raw confusion matrix comparing predicted panels to actual panels under LOOCV; the Control and early Lyme panels are strongly dominated by the diagonal (correct) entries, while predictive performance is more equivocal for Lyme Arthritis and PTLDS. These data strongly suggest that immunoglobulin class and IgG isotyping are likely more helpful to distinguish early Lyme disease cases. Comparing the multivariable models to single predictor models, we generally found that the multivariable models outperformed the others substantially, except for the multinomial-logit type models. More than half of sera panels were perfectly identified under cross validation by the random forest model (56%), while 38%, 32%, 28%, 28%, and 26% were identified for IgG1, IgG3, IgM, IgA1, and IgG4 by single predictor models, respectively. Broadening the definition of accuracy to focus just on Lyme vs controls, we see high accuracy across all methods (Table S1). For comparison, tests of association between each pair of sera panels with respect to each immunoglobulin are summarized in Tables S2 to S6.

**TABLE 2:** Predictive performance under LOOCV by model^*a*^

Measure	Panel by LD stage	Multinomial regression	glmnet	xgboost	SVM	Single tree	Random forest
Sensitivity	Control	0.93	1.00	0.93	0.93	0.95	0.93
	Early Acute <1 mo	0.66	0.44	0.56	0.56	0.53	0.72
	Early Acute >1 mo	0.50	0.54	0.77	0.77	0.58	0.69
	Early Convalescent	0.56	0.33	0.46	0.46	0.44	0.38
	Late Lyme arthritis	0.11	0.06	0.22	0.22	0.17	0.17
	PTLDS	0.12	0.00	0.12	0.12	0.08	0.16
Specificity	Control	0.94	0.70	0.95	0.95	0.94	0.94
	Early Acute <1 mo	0.95	0.97	0.95	0.95	0.87	0.92
	Early Acute >1 mo	0.93	0.91	0.90	0.90	0.88	0.93
	Early Convalescent	0.74	0.76	0.77	0.77	0.85	0.79
	Late Lyme arthritis	0.95	0.98	0.97	0.97	0.94	0.95
	PTLDS	0.93	1.00	0.92	0.92	0.92	0.92
PPV	Control	0.80	0.49	0.84	0.84	0.81	0.82
	Early Acute <1 mo	0.75	0.74	0.69	0.69	0.47	0.66
	Early Acute >1 mo	0.54	0.50	0.56	0.56	0.45	0.62
	Early Convalescent	0.38	0.28	0.36	0.36	0.45	0.34
	Late Lyme arthritis	0.20	0.25	0.44	0.44	0.25	0.27
	PTLDS	0.21	NA	0.20	0.20	0.14	0.25
NPV	Control	0.98	1.00	0.98	0.98	0.98	0.98
	Early Acute <1 mo	0.93	0.89	0.91	0.91	0.90	0.94
	Early Acute >1 mo	0.92	0.92	0.96	0.96	0.93	0.95
	Early Convalescent	0.86	0.80	0.84	0.84	0.85	0.82
	Late Lyme arthritis	0.91	0.90	0.92	0.92	0.91	0.91
	PTLDS	0.87	0.86	0.87	0.87	0.86	0.87

^*a*^ Legend: LD, Lyme disease; PTLDS, post treatment Lyme disease syndrome; mo, month; PPV, positive predictive value; NPV, negative predictive value.

**TABLE 3:** Predicted vs observed panels from sera data—random forest

		Observed					
		Control	Early Conv	Early Acute >1 mo	Early Acute <1 mo	LA	PTLDS
Predicted	Control	37	2	0	1	0	5
	Early Conv	3	15	4	2	9	11
	Early Acute >1 mo	0	4	18	5	1	1
	Early Acute <1 mo	0	5	3	23	2	2
	LA	0	4	1	1	3	2
	PTLDS	0	9	0	0	3	4

## DISCUSSION

The type of antigen (protein, carbohydrate, nucleic acid, lipid) binding to the B cell receptor triggers a signaling cascade that ultimately results in B cell proliferation, differentiation, and production of immunoglobulin/antibody. T-cell independent stimulation of B cells induces differentiation into short-lived plasma cells with limited class switching, whereas T-cell-dependent stimulation permits affinity maturation, class switching to the entire array of immunoglobulin classes available, and differentiation into long-lived memory B cells (11). The order of switching between the immunoglobulin heavy chain (Fc) is time dependent, progressing from IgM/D to IgG_3_/IgG_1_/IgA_1_/IgG_2_ /IgG_4_ and later to IgE/IgA_2_ (11, 12). This allows for the B cell to produce antigen-specific immunoglobulins with different biologic effector functions. Based on these fundamental principles, we hypothesized that we may distinguish disease stage by targeting the four classes of immunoglobulin (Ig M/D/G/A) and all four IgG isotypes (IgG 1/2/3/4) specific to *B. burgdorferi* VlsE in serum obtained from Lyme disease patients with supporting serological evidence of microbiological infection. In this study, we show that *B. burgdorferi*-VlsE-specific antibody switching proceeds in a predictable sequence between immunoglobulin classes and IgG isotype as Lyme disease progresses from early to late stages.

IgM antibodies are the first immunoglobulin expressed during B cell development, have low-avidity for antigen (are poly-reactive, less specific), and function by opsonizing antigen for destruction and fixing complement; IgM is frequently used to diagnose acute exposure to a pathogen (11) including Lyme disease (7, 24). As expected, IgM was detected at the earliest, acute stage of Lyme disease (Fig. 2 and 3).

Circulating IgD is found at low levels in serum and has a short half-life, and its function is not clear as it is not known to participate in effector mechanisms. However, there is an FcR for IgD (FcδR) in CD3 T cells. Thus, it has been proposed that IgD might serve as a bridge for antigen presentation by B cells to T cells (11). The presence of transient IgD in serum could be indicative of a pre-adaptive immune response ongoing in the early stages of Lyme disease when mature B cells producing IgM+/IgD+ reach the spleen. We evaluated the presence of total IgD and VlsE-specific IgD in blood collected at different Lyme disease stages (Fig. S1). Although we observed increased prevalence of total IgD in one panel of serum from patients classified as early Lyme disease, our VlsE-specific analyses were inconclusive. This subject requires further experimentation.

When we performed a thorough analysis of immunoglobulin class (M, G, A) and IgG isotypes to VlsE broken by Lyme disease stage, we found that the Early Acute Lyme disease panels contained the highest variety of immunoglobulin, ranging from IgM to IgG3/IgG1 to IgA1, whereas Early Convalescent, Lyme Arthritis, and PTLDS were enriched mostly for IgG1 (Fig. 2). Specifically, IgG3 and IgA1 associated mostly with Early Acute <1 month (63% and 72%, respectively) and Early Acute >1 month (92% and 85%, respectively), IgG1 associated with all clinically defined stages of Lyme disease (91%–100%) as well as 76% of PTLDS and that IgG4 associated mostly with Lyme arthritis (56%) (Fig. 3). Others have also shown that IgG3 is associated with early stages of Lyme disease, whereas IgG4 is associated with Lyme arthritis and post-antibiotic Lyme arthritis (34–37). An association between elevated levels of *B. burgdorferi* antigen-specific IgA and particular disease manifestations was found in some patients with early to early disseminated LD (38). IgG1 and IgG3 bind complement, but IgG4 does not. It is well established that killing of *B. burgdorferi* is an antibody-mediated complement-dependent and complement-independent process (39, 40). Furthermore, IgG1, IgG3, and IgA1 are generally induced by protein antigens (which are likely to be present in the earliest stages of infection) and IgG3/IgG1 are activating antibodies that bind all three Fcγ receptors (FcγR I, II, III) on the surface of effector cells such as natural killer and macrophages (11, 12, 37, 41). IgG3 is the first IgG to appear in serum as switching from IgM/D to IgG takes place and IgG3 are early effectors dependent of T cell help (12). Furthermore, IgG3 are potent inducers of antibody-dependent cell cytotoxicity (11) and the high detection of *B. burgdorferi*-specific IgG3 in patients with localized erythema migrans (37) suggests that a T cell-dependent cytotoxic response may be engaged in the earliest stages of Lyme disease.

Although IgG4 can be induced by repeated exposure to protein antigen, IgG4 and IgG2 are generally induced by polysaccharide antigens and IgG responses to bacterial capsular antigens can be almost completely restricted to IgG2; this subclass is mostly comprised of anti-carbohydrate antibodies (12). Early in the course of infection, in the absence of T-cell help, polysaccharide antigens may induce class switching to IgG2 (12). Given the association of peptidoglycan (PG) as a retained antigen in Lyme arthritis (42) and the likelihood that early Lyme disease would be associated with higher levels of peptidoglycan muramyl dipeptides, if PG were our antigen instead of VlsE, we speculate that IgG2 may have been increased. IgG2 binds only to FcγR IIa; production of IgG2 is associated with IgG4 and IgE which suggests long-term exposure to antigen. IgG4 antibodies bind to FcγR II & III and are often formed following repeated exposure to antigen in a non-infectious setting (12). Switching to IgG4 may be modulated by interleukin 10, linking this subclass to induction of tolerance (43). Thus, the association of IgG4 to Lyme arthritis suggests that dysregulated immune responses in the joint may ensue due to the presence of non-infectious antigenic remnants of *B. burgdorferi* (44).

The blinded panel contained very few positive immunoglobulin samples, including IgM (Fig. 2 and 3). This is due to patients being at a very early stage of infection with antibody response not yet detectable and that some were not infected with *B. burgdorferi* as per inclusion criteria. PTLDS showed a profile more alike convalescent Lyme disease than the other stages.

The multivariable models are promising (Fig. 4; Tables 2 and 3; Table S1). Both random forests and individual decision trees do a remarkably good job of classifying sera panels, particularly those for which the antibodies provide strong signals. The differential performance between these two methods (ensemble random forests and individual trees) is lower than expected. For the Early Acute Lyme disease serum, the random forest and other ensemble classifiers are substantially more sensitive. Notably, none of the techniques used here are particularly high dimensional, and neither would they generally be considered “deep learning.” With larger samples, however, there are numerous opportunities for the expansion and improvement of these methods. These include the exploration of other classifiers like deep neural networks. Nevertheless, practical concerns may limit the availability of larger numbers of samples or higher dimensional antibody profiles, and some ambiguity remains concerning the classification of individual panels along the exposure/disease course in the absence of a gold standard measure of progression. Nevertheless, these initial results highlight a promising approach for future investigation. In our models, we targeted only the VlsE antigen; we expect that targeting other *B. burgdorferi* antigens such as FlaB, OspC, ErpB, DbpA, p28 (14, 15), peptidoglycan (42), in addition to VlsE, may contribute more antigen-specific immunoglobulin isotypes to power our model and make more accurate predictions of disease stage.

### Limitations of this study

They include testing samples in duplicate for technical replicates, the use of a laboratory-developed VlsE1-ELISA and not using a clinically defined, serologically unclassified set of samples for validation of the assay. Testing in duplicate was done due to the limited volume of serum available, the large number of antibody classes and isotypes to evaluate using the same reagents for all plates, and the potential for cross-contamination due to manual distribution of the samples. Our home-brew assay was not ideal, but it was appropriate for the initial discovery testing reported in this study. As such, analytical sensitivity, analytical specificity, and reproducibility of large panels of clinical data were not assessed. The evidence presented in this study would be stronger if validation had been done using a clinically defined, serologically unclassified or blinded set of samples. We initially included the blinded Lyme Disease Biobank 2016 panel for this purpose. However, given that it contained very few seropositive samples and that it included samples from healthy individuals from endemic areas, the panel was not appropriate for validation.

In summary, our results are novel in identifying IgG isotypes as potential biomarkers of Lyme disease stage, and there may be diagnostic value in quantifying antibody classes and isotypes in serum from Lyme disease patients. We also found that immunoglobulin class and IgG isotyping is likely more helpful to distinguish early Lyme disease cases. We envision a multiplexed clinical diagnostic test that detects a range of immunoglobulin classes and IgG isotypes to VlsE and to other *B. burgdorferi* antigens (OspC, DbpA, FlaB), with results being interpreted using AI to suggest the likely disease stage, which could be correlated with clinical findings to increase or decrease confidence in the diagnosis. Or alternatively, the study’s findings can be used to select isotypes that are present most frequently across certain disease stages with the idea of focusing on those isotypes for refinement of diagnostic assays. Future studies could include analysis of serially collected samples at different timepoints over the course of infection, as it may be useful to know how antimicrobial treatment may affect the characteristic succession of immunoglobulin class and isotope switching.

## Data Availability

ELISA optimization and data source files are provided in Supplemental material.

## SUPPLEMENTAL MATERIAL

Supplemental figure and tables jcm.00347-25-s0001.docx Fig S1 and Tables S1 to S6.

Suplemental material source files for Fig 2 and Fig 3 jcm.00347-25-s0002.xlsx Fig. S2 and S3.

Supplemental material jcm.00347-25-s0003.xlsx Optimization for secondary antibody dilution.

ASM does not own the copyrights to Supplemental Material that may be linked to, or accessed through, an article. The authors have granted ASM a non-exclusive, world-wide license to publish the Supplemental Material files. Please contact the corresponding author directly for reuse.
