# Supplementary material for: Class and isotype of VlsE-specific antibody differentiates Lyme disease stage
Source: J Clin Microbiol. 2025 Jul 15;63(8):e00347-25. doi: 10.1128/jcm.00347-25 (PMC12345165; doi:10.1128/jcm.00347-25)
Supplement: Supplemental figure and tables — Fig S1 and Tables S1 to S6. [file jcm.00347-25-s0001.docx]

**SUPPLEMENTAL MATERIAL**

Figure S1. Total IgD in a panel of Lyme disease serum (CDC). Nr of patients (prevalence) with more than 10ng IgD/mL of serum: n=5 Healthy, n=25 Early Lyme, n= 10 Late Lyme.

| **Table S1: Overall Performance: All Cases vs. Controls** | | | | |
| --- | --- | --- | --- | --- |
| **Model** | **Sensitivity** | **Specificity** | **PPV** | **NPV** |
| Multinomial Reg. | 0.94 | 0.93 | 0.98 | 0.80 |
| glmnet | 0.70 | 1.00 | 1.00 | 0.49 |
| xgboost | 0.95 | 0.93 | 0.98 | 0.84 |
| SVM | 0.95 | 0.93 | 0.98 | 0.84 |
| Single tree | 0.94 | 0.95 | 0.98 | 0.81 |
| Random Forest | 0.94 | 0.93 | 0.98 | 0.82 |

Legend: Multinomial Reg, multinomial regression; glmnet, penalized multinomial regression model; xgboost, gradient boosted tree model; SVM, support vector machine model; PPV, positive Predictive Value; NPV, Negative Predictive Value

| **Table S2: IgA1 Pairwise Wilcoxon Rank Sum p-values** | | | | | |
| --- | --- | --- | --- | --- | --- |
|  | **Control** | **Early**  **Convalescent** | **Early Acute > 1 mo** | **Early Acute < 1 mo** | **LA** |
| **Early Convalescent** | 0.0003 |  |  |  |  |
| **Early Acute > 1 mo** | <0.0001 | 0.0002 |  |  |  |
| **Early Acute < 1 mo** | <0.0001 | 0.0021 | 0.4748 |  |  |
| **LA** | 0.0099 | 0.6856 | 0.0005 | 0.0043 |  |
| **PTLDS** | 0.0023 | 0.5904 | <0.0001 | 0.0003 | 0.9515 |

Legend: LA, Lyme Arthritis; PTLDS, Post Treatment Lyme Disease Syndrome; mo, month

| **Table S3: IgG1 Pairwise Wilcoxon Rank Sum p-values** | | | | | |
| --- | --- | --- | --- | --- | --- |
|  | **Control** | **Early**  **Convalescent** | **Early Acute > 1 mo** | **Early Acute < 1 mo** | **LA** |
| **Early Convalescent** | <0.0001 |  |  |  |  |
| **Early Acute > 1mo** | <0.0001 | <0.0001 |  |  |  |
| **Early Acute < 1mo** | <0.0001 | 0.9555 | <0.0001 |  |  |
| **LA** | <0.0001 | 0.0751 | 0.0991 | 0.0660 |  |
| **PTLDS** | <0.0001 | 0.8101 | <0.0001 | 0.9555 | 0.0660 |

Legend: LA, Lyme Arthritis; PTLDS, Post Treatment Lyme Disease Syndrome; mo, month

| **Table S4: IgG3 Pairwise Wilcoxon Rank Sum p-values** | | | | | |
| --- | --- | --- | --- | --- | --- |
|  | **Control** | **Early Convalescent** | **Early Acute > 1 mo** | **Early Acute < 1 mo** | **LA** |
| **Early Convalescent** | <0.0001 |  |  |  |  |
| **Early Acute > 1 mo** | <0.0001 | <0.0001 |  |  |  |
| **Early Acute < 1 mo** | <0.0001 | 0.1525 | <0.0001 |  |  |
| **LA** | <0.0001 | 0.2378 | 0.0005 | 0.8180 |  |
| **PTLDS** | 0.0278 | 0.0864 | <0.0001 | 0.0078 | 0.0126 |

Legend: LA, Lyme Arthritis; PTLDS, Post Treatment Lyme Disease Syndrome; mo, month

| **Table S5: IgG4 Pairwise Wilcoxon Rank Sum p-values** | | | | | |
| --- | --- | --- | --- | --- | --- |
|  | **Control** | **Early Convalescent** | **Early Acute > 1 mo** | **Early Acute < 1 mo** | **LA** |
| **Early Convalescent** | 0.0937 |  |  |  |  |
| **Early Acute > 1 mo** | <0.0001 | 0.0021 |  |  |  |
| **Early Acute < 1 mo** | 0.0085 | 0.4152 | 0.0073 |  |  |
| **LA** | 0.0002 | 0.0065 | 0.4152 | 0.0085 |  |
| **PTLDS** | 0.7822 | 0.3425 | 0.0004 | 0.0992 | 0.0032 |

Legend: LA, Lyme Arthritis; PTLDS, Post Treatment Lyme Disease Syndrome; mo, month

| **Table S6: IgM Pairwise Wilcoxon Rank Sum p-values** | | | | | |
| --- | --- | --- | --- | --- | --- |
|  | **Control** | **Early Convalescent** | **Early Acute > 1 mo** | **Early Acute < 1 mo** | **LA** |
| **Early Convalescent** | 0.0011 |  |  |  |  |
| **Early Acute > 1 mo** | <0.0001 | <0.0001 |  |  |  |
| **Early Acute < 1 mo** | <0.0001 | <0.0001 | 0.8584 |  |  |
| **LA** | 0.0001 | 0.5968 | <0.0001 | <0.0001 |  |
| **PTLDS** | 0.0781 | 0.3588 | <0.0001 | <0.0001 | 0.2271 |

Legend: LA, Lyme Arthritis; PTLDS, Post Treatment Lyme Disease Syndrome; mo, month
